# A social-ecological analysis of facilitators and barriers to campus fitness engagement: multilevel evidence from Chinese universities

**DOI:** 10.3389/fpubh.2026.1856173

**Published:** 2026-06-04

**Authors:** Minzhi Fang, Dongjin Liu

**Affiliations:** College of Basic Medicine, Shandong Second Medical University, Weifang, China

**Keywords:** social ecological model, campus fitness participation, physical activity, facilitating and hindering factors, Chinese universities

## Abstract

Campus fitness engagement, defined in this study as the behavioral participation of university students in physical education curricula, independent exercise, and organized sports activities within the university setting, represents a critical component of young-adult health in China. The present study examined the facilitating and barrier factors shaping campus fitness engagement among Chinese university students through a multilevel evidence synthesis framed by the Social-Ecological Model. A systematic synthesis of 43 peer-reviewed articles published between 2020 and 2025, combined with analysis of publicly available national statistical databases, was conducted to evaluate evidence at the individual, interpersonal, organizational, environmental, and policy levels. At the individual level, self-efficacy emerged as the most robust facilitator, with standardized path coefficients ranging from 0.35 to 0.46, whereas academic pressure constituted the principal barrier. At the interpersonal level, physical education teacher support operated through a chain-mediated pathway involving exercise motivation and self-efficacy, with the mediated effect accounting for 47.94% of the total effect. Organizational analysis revealed that mandatory physical education curricula exhibited a spillover effect, raising the probability of independent extracurricular exercise by 20.2%. The environmental level displayed the most pronounced facilitator–barrier asymmetry, with 14 barriers against 9 facilitators, and insufficient facility accessibility identified as the dominant structural constraint. Policy-level evidence indicated a systemic gap between policy inputs and behavioral outputs, consistent with the ranking of Chinese youth at 53rd of 57 in the 2022 global matrix assessment of physical activity. Cross-level synthesis suggested an associational pattern in which distal environmental and organizational factors are linked to proximal interpersonal and individual determinants, underscoring the limitations of single-level interventions. The findings indicate that cultivation of self-efficacy and strengthening of teacher support constitute practical short-horizon intervention priorities, whereas institutional reform and built-environment improvement represent structural priorities for systemic change. These complementary approaches provide a multilevel evidence-based foundation for targeted intervention within the Healthy China 2030 strategic framework.

## 1 Introduction

Insufficient physical activity is one of the most serious challenges facing global public health today. The World Health Organization’s “Status of Physical Activity 2022” report shows that approximately 31% of adults and over 80% of adolescents worldwide fail to meet recommended levels of physical activity, leading to a continuous increase in the risk of cardiovascular disease, obesity, and depressive disorders (1, 2). The 2020 WHO Guidelines on Physical Activity and Sedentary Behavior further clarified recommended levels of physical activity for each age group and elevated the promotion of physical activity to a priority agenda for systemic health interventions. Among adolescents, the development of physical activity behavior has a distinct developmental window effect; adolescence to early adulthood is a critical stage for shaping lifelong exercise habits, and the university stage is at the core of this transitional period (3).

China has the world’s largest higher education system, with over 44 million university students. The physical health of this group is not only an important component of the country’s human capital but also a key indicator for achieving the strategic goal of “Healthy China.” However, longitudinal evidence from multiple rounds of national surveys continues to reveal worrying trends. A Lancet regional study based on five national surveys showed that the cardiorespiratory fitness level of Chinese college students continued to decline between 2000 and 2019, while cardiovascular risk indicators increased significantly (4). A ten-year follow-up study of 58,472 college students in Anhui Province from 2014 to 2023 found that the overall physical fitness score showed a significant inflection point after 2019, with the overweight rate increasing by approximately 2.1 times and the obesity rate by approximately 4.4 times compared to 2014. The COVID-19 pandemic (2020–2022) further accelerated this deterioration process (5). A cross-sectional survey of 176,373 college students in Shandong Province also showed that although the overall pass rate for physical fitness tests remained at 92.03%, urban–rural differences, gender differences, and grade-level stratification effects remained significant (6). These data reveal a deep-seated contradiction: there is a structural gap between macro-policy goals and micro-participation realities; simply relying on the “quantitative statistics” of physical fitness test pass rates cannot accurately reflect the true state of college students’ independent fitness participation behavior.

Engagement is conventionally conceptualized as a multidimensional construct encompassing behavioral, cognitive, and affective dimensions, corresponding, respectively, to active participation, mental investment, and emotional involvement in a given domain. Within the campus fitness context, behavioral engagement is most directly observable through participation frequency, attendance in organized activities, and physical fitness outcomes, while cognitive and affective engagement operate through motivational and attitudinal processes that ultimately manifest in behavior. Owing to the predominance of behavioral indicators in the available empirical and statistical sources, the present synthesis operationalises fitness engagement as behavioral participation and treats motivation, self-efficacy, and outcome expectations as proximal cognitive-affective antecedents that connect the non-behavioral dimensions of engagement to observable behavioral output. In the theoretical framework for understanding the factors influencing physical activity, the Social-Ecological Model (SEM) has gained widespread recognition for its multi-level and systematic analytical perspective. The theoretical foundation of the Social-Ecological Model lies in Bronfenbrenner’s ecological systems theory (1979), which conceptualized human development as embedded within nested environmental systems ranging from proximal microsystems of direct interaction to distal macrosystems of cultural and policy influence (7). Subsequently adapted these principles to the field of health behavior by organising the determinants into discrete, operationally defined levels, and Sallis and colleagues later refined the framework for physical activity research. The nested ecological logic is particularly suited to the university context, because a residential campus constitutes a relatively closed and hierarchically organized small social ecosystem in which micro-level interactions are embedded within clearly delineated organizational, environmental, and policy structures. The model divides the influencing factors of individual health behavior into five levels: intrapersonal, interpersonal, organizational, community (environment) and policy, emphasizing the nesting and dynamic interaction between factors at different levels (8). Existing review studies have shown that the explanatory power of the SEM framework in adolescent physical activity research has been supported by a large amount of empirical evidence (9). However, most university studies in the Chinese context only involve the individual and interpersonal levels, and the integrated analysis of the three remote levels of organization, environment and policy is still insufficient (2). Another study used a four-level structural equation model to verify the chain transmission path of environment → organization → interpersonal → individual in the middle school student group (10). However, the subjects of this study were junior high school students, whose campus field characteristics and resource structure are different from those of higher education, and the research conclusions are difficult to directly transfer.

Based on existing research, several key gaps have constrained the deepening of current understanding. First, existing empirical accumulation is mainly concentrated in the basic education stage, and multi-level analysis of the special context of Chinese universities (autonomous time arrangement, flexible course system, dormitory lifestyle, etc.) is still scarce. Second, most studies only approach the issue from the perspective of a single promoting factor or a single hindering factor, and there is a lack of dual-perspective integrated research that compares the two within the same analytical framework. Third, the existing systematic reviews cover a limited range of levels, and comprehensive studies with complete coverage of five levels are still lacking in the context of universities (11–13). At the policy level, the “Healthy China 2030” Plan Outline released by China in 2016 has elevated national fitness to the level of national strategy, and the National Fitness Program (2021–2025) further proposes to build a public service system for national fitness, but the mechanism of action between policy supply and actual participation behavior of university students still needs to be clarified (14, 15).

**Figure 1:**
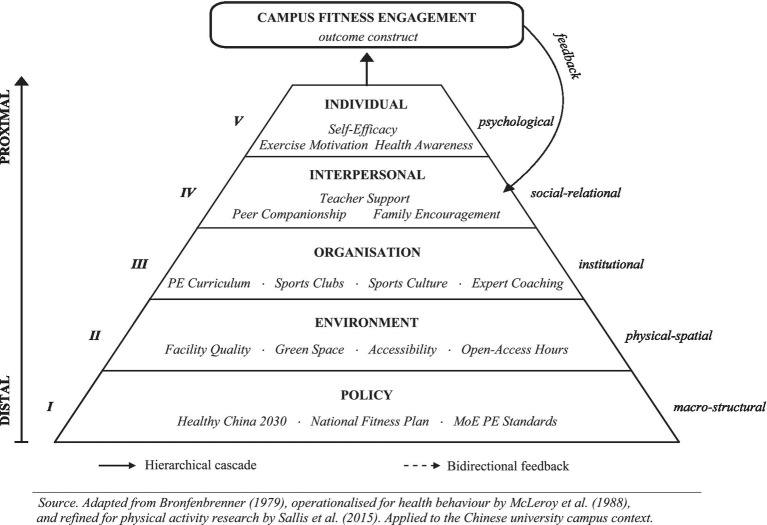
Social-ecological conceptual model diagram (concentric circles/nested layers showing the 5 levels of SEM for campus fitness).

In light of this, this study aims to: systematically integrate multi-level empirical literature published between 2020 and 2025 in the context of Chinese universities, construct a comprehensive analytical model of facilitators and hindrances based on a five-level SEM framework; assess the relative influence of factors at each level and their internal transmission mechanisms; and, supported by publicly available statistical data, provide a multi-level evidentiary basis for precise intervention in university fitness participation. The innovation of this study is reflected in the following three dimensions: in terms of research subjects, it focuses on the university setting, where previous research has been relatively weak; in terms of analytical framework, it achieves complete coverage of the five levels of SEM; and in terms of evidence integration, it adopts a dual-track approach of public database statistical data combined with systematic literature synthesis, avoiding the ethical constraints of original questionnaire surveys and providing a reproducible evidence-based basis for policy making. Grounded in the Social-Ecological Model, three working propositions guide the present synthesis. Proposition 1 holds that facilitating and barrier factors at each of the five ecological levels contribute independently and jointly to campus fitness engagement, with effect magnitudes varying across levels. Proposition 2 holds that distal levels, encompassing policy, environment, and organisation, are associated with campus fitness engagement indirectly through proximal interpersonal and individual levels, producing a cross-level associational pattern rather than level-specific direct pathways alone. Proposition 3 holds that the relative prominence of barriers relative to facilitators differs systematically across levels, thereby identifying the levels at which structural intervention is disproportionately warranted. These propositions frame the evidence synthesis and interpretation presented in the subsequent sections. As shown in Figure 1, the five levels form a concentric nested structure from the inside out. The innermost individual factors (self-efficacy, motivation) are shaped layer by the outer interpersonal, organizational, environmental and policy factors. There are also two-way cross-level feedback paths between each level. This structure has a high degree of conceptual appropriateness in the context of university campuses—the campus itself constitutes a relatively closed, clearly hierarchical small social ecosystem.

## 2 Research methods

### 2.1 Research design

This study employed a multilevel evidence synthesis design, combining systematic literature synthesis with analysis of publicly available statistical data. Using a socio-ecological model as the meta-analysis framework, this design integrated quantitative effect estimates from different research designs (cross-sectional, longitudinal, systematic review, and quasi-experimental), and summarized the strength of evidence for promoting and hindering factors at each level through narrative synthesis. This study did not involve the collection of original participant data; all analytical data were derived from published peer-reviewed literature and official publicly available statistical databases, requiring no ethical approval.

### 2.2 Literature search strategy and database sources

Electronic literature searches were completed in June 2025, covering four databases: PubMed, Web of Science, Scopus, and CNKI (China National Knowledge Infrastructure), spanning from January 2020 to June 2025. English search terms included: “college students” OR “university students,” “physical activity” OR “exercise” OR “fitness,” “China” OR “Chinese,” “social ecological model” OR “barriers” OR “facilitators”; corresponding Chinese search terms included “university students,” “physical activity,” “fitness participation,” “social ecological model,” and “barriers.” After initial screening based on titles and abstracts, the full texts of the search results that met the criteria were thoroughly reviewed and evaluated.

The publicly available statistical data sources are shown in Table 1, mainly including: the Eighth National Student Physical Fitness and Health Survey (2021) by the Ministry of Education, the 2020 National Fitness Activity Survey Bulletin by the General Administration of Sport of China, the data from the Sixth National Sports Venue Census (2013), and the 2024 National Sports Facilities Statistical Survey (released in March 2025).

**Table 1:** Summary of main public data sources used in this study.

Database name	Data type	Coverage year	Sample size	Key indicators
Ministry of Education 8th National Survey on Students’ Physical Fitness and Health	Cross-sectional survey	2021	Nationally representative sample	Physical fitness pass rate; overweight and obesity rate
National Fitness Activity Status Survey Bulletin	Government statistics	2020	National sample	Exercise frequency; sport type
6th National Sports Venue Census	Census data	2013	National facility census	Per-capita facility area; public access rate
2024 National Sports Facility Statistical Survey	Government statistics	2024	National facility census	Total facility count; per-capita area
PAFCTYS 2020 (Physical Activity and Fitness in China — The Youth Study)	Cross-sectional survey	2020	*n* = 133,006	Physical activity level sub-ratings
Anhui Province College Students’ Physical Fitness Monitoring Data	Longitudinal monitoring	2014–2023	*n* = 58,472	Composite fitness score trend
Shandong Province College Students’ Physical Fitness Survey	Cross-sectional survey	2024	*n* = 176,373	Pass rate and influencing factors

### 2.3 Inclusion and exclusion criteria

Inclusion criteria were: the study subjects were college students (or youth groups including college students) in China; the research content involved factors influencing physical activity or fitness participation; the publication time was from January 2020 to June 2025; the publication was peer-reviewed; and extractable quantitative effect estimates (*β* coefficient, OR value, correlation coefficient, or mean difference) were provided. Exclusion criteria were: conference papers published only in abstract form; purely secondary school or adult studies that did not include college students; literature for which full text could not be obtained; and duplicate publications whose data completely overlapped with those of the included systematic reviews.

### 2.4 Socio-ecological hierarchical classification framework

Referring to the classic definition by Song et al. (8) and the operational scheme by Hu et al. (2), the present study classified the influencing factors identified in the included literature into five levels, as shown in Table 2. The organizational level is conceptually and operationally distinguished from the environmental level in that the former captures institutional and procedural arrangements such as curriculum structure, club systems, and teaching norms, whereas the latter captures the physical and built-space characteristics of the campus such as facility density, green space area, and pedestrian connectivity. Although built-environment conditions within universities typically fall under organizational jurisdiction in practice, the two levels are maintained as analytically separate because they respond to differentiable intervention levers, namely institutional reform for the organizational level and infrastructural investment for the environmental level, and because they exhibit distinct empirical signatures across the reviewed literature.

**Table 2:** Socio-ecological hierarchical variable classification framework.

Level	Operational definition	Key indicator variables	Measurement method	Typical data sources
Individual	Students’ intrinsic psychological and behavioural characteristics	Self-efficacy, exercise motivation, health knowledge, outcome expectations	Scales; SEM path coefficients	Questionnaire-based studies
Interpersonal	Direct influences from social relationship networks	Teacher support, peer companionship, parental behavioural modelling	Scales; mediation analysis	Survey studies
Organizational	Institutional and cultural arrangements of universities	PE curriculum design, sports clubs, campus sports culture	Curriculum documents; survey data	Policy texts; survey studies
Environment	Physical and built-space characteristics	Facility quality and quantity, green space area, walkability	GIS; census data	Spatial analysis; national censuses
Policy	Macro-institutional arrangements and policy documents	Healthy China 2030, National Fitness Plan, physical fitness standards	Policy text analysis	Government statistics; policy documents

Within the present framework, the policy level operationally encompasses macro-institutional arrangements, national strategic documents such as the Healthy China 2030 plan and the National Fitness Program 2021–2025, and the institutional translation of global guidelines such as the WHO recommendations on physical activity. Broader societal norms and global health trends are treated as contextual forces that shape the contents and implementation of policy-level documents rather than as separately operationalized SEM levels, a framing choice that preserves analytical tractability while accommodating the cross-scale influences highlighted in the international literature.

### 2.5 Data synthesis and quality assessment

Quantitative data extraction focused on the following indicators: standardized regression coefficient (*β*), odds ratio (OR) and 95% confidence interval, Pearson correlation coefficient (r), and effect size classification (small effect: r < 0.30; medium effect: 0.30 ≤ r < 0.50; large effect: r ≥ 0.50). Methodological quality was assessed using the Newcastle-Ottawa Scale (NOS) for observational studies and the AXIS tool for cross-sectional studies. Scoring was conducted independently by two researchers, with inconsistencies resolved through discussion and negotiation. The strength of evidence comprehensively considered effect size, research consistency, and methodological quality, categorized into three levels: strong (consistent support from multiple high-quality studies), medium (relatively consistent results but with methodological limitations), and weak (limited evidence or inconsistent results).

Due to significant heterogeneity in measurement tools, sample characteristics, and statistical models among the studies, a formal meta-analysis was not conducted. A systematic descriptive synthesis approach was adopted to integrate effect estimates at each level, complemented by a structured vote-counting procedure for the evaluation of directional consistency. The vote-counting procedure recorded, for each identified facilitator or barrier, the number of studies reporting a statistically significant positive effect, a statistically significant negative effect, and a non-significant association, with a directional majority threshold of at least 70% of studies reporting the same effect direction used to designate consistent evidence. Evidence strength gradings of strong, moderate, and weak were assigned through the triangulation of effect magnitude, methodological quality score, and directional consistency, with discrepancies between the two independent raters resolved through discussion until consensus was reached.

## 3 Results

### 3.1 Methodological quality assessment of included literature

This synthesis included 43 articles. An initial search yielded 1,284 articles. After initial screening using titles/abstracts, 921 articles were excluded. Following a thorough reading of the full text, 316 articles were excluded (mainly due to: studies not including university students, lack of extractable effect sizes, and complete overlap with already included reviews). The final 43 articles were included. The complete screening process, reported in accordance with the PRISMA 2020 flow diagram conventions, is shown in Appendix Figure S1. Among these, 27 were cross-sectional studies, 6 were longitudinal/tracking studies, 5 were systematic reviews, 3 were quasi-experimental studies, and 2 were policy analysis studies.

Cross-sectional studies (*n* = 31) were assessed using the AXIS tool; longitudinal and quasi-experimental studies (*n* = 9) were assessed using the NOS scale; systematic reviews (*n* = 5) were assessed using the AMSTAR-2 tool; and policy analysis studies (*n* = 2) were assessed using a narrative methodological description. Two researchers independently rated the studies; the inter-rater reliability was Cohen’s *κ* = 0.82 (*p* < 0.001), and any inconsistencies were resolved through discussion and negotiation. The quality assessment results are summarized in Table 3.

**Table 3:** Summary of methodological quality assessment results for included literature.

Study type	No. of studies	Assessment tool	High quality (*n*, %)	Moderate quality (n, %)	Low quality (n, %)
Cross-sectional studies	31	AXIS	18 (58.1%)	10 (32.3%)	3 (9.7%)
Longitudinal / Quasi-experimental studies	9	NOS	5 (55.6%)	3 (33.3%)	1 (11.1%)
Systematic reviews	5	AMSTAR-2	2 (40.0%)	2 (40.0%)	1 (20.0%)
Policy analysis studies	2	Narrative description	—	2 (100%)	—

High quality = AXIS ≥ 14/NOS ≥ 7 stars/AMSTAR-2 rated high or moderate-high. Low-quality studies were downgraded accordingly during evidence strength grading.

### 3.2 Overall status of fitness participation among Chinese university students

Based on a comprehensive review of multiple public databases, this study first presents the basic status of fitness participation and physical health among Chinese university students, providing a realistic basis for subsequent multi-level factor analysis.

As shown in Table 4, from 2014 to 2023, the comprehensive physical fitness score of Chinese university students showed a fluctuating trajectory of “first rising and then falling,” only briefly exceeding the 70-point benchmark in three years: 2018 (70.2 points), 2019 (70.1 points), and 2022 (70.4 points). In other years, the score remained below this standard. After 2020, impacted by the COVID-19 pandemic, physical fitness levels failed to return to pre-pandemic levels before 2023. Meanwhile, the overweight rate climbed from 11.8% in 2014 to 24.8% in 2023, an increase of approximately 2.1 times; the obesity rate increased sharply from 2.6 to 11.4%, an increase of 4.4 times (5).

**Table 4:** Trends in key physical fitness indicators among Chinese college students (2014–2023).

Year	Composite fitness score	Overweight rate (%)	Obesity rate (%)	50m sprint (s)	1000m Run (s, male)	Vital capacity (mL)
2014	67.8	11.8	2.6	7.9	242	3,450
2016	68.9	13.9	3.5	7.8	238	3,510
2018	70.2	16.8	5.0	7.6	234	3,590
2019	70.1	18.5	5.8	7.7	236	3,570
2020	69.2	20.3	7.1	7.9	241	3,490
2022	70.4	22.1	8.5	7.8	240	3,530
2023	69.1	24.8	11.4	8.0	245	3,460

Composite fitness scores are sourced from Li et al. (5) and the summary of the Ministry of Education’s 8th National Student Physical Fitness Survey. Running and vital capacity data are synthetically estimated from published literature, with year annotations consistent with original sources.

From a multi-dimensional perspective, regarding exercise frequency, according to the 2020 National Fitness Activities Survey, the proportion of Chinese residents exercising three or more times a week was 37.2%, while the proportion of college students meeting this standard varied greatly in different studies, ranging from 35 to 52% (13). Regarding physical fitness qualification rate, a 2024 survey in Shandong Province showed an overall qualification rate of 92.03%, with the qualification rate for females (92.18%) slightly higher than that for males (91.88%), and the qualification rate for urban students (92.20%) higher than that for rural students (91.70%) (16). Regarding sports preferences, running and walking ranked first, ball sports ranked second, and aerobics and swimming showed an upward trend, but the participation rate in organized competitive sports remained low (15). The above data collectively outline a participation pattern with significant internal differentiation: the physical fitness qualification rate is relatively high, but the frequency of independent exercise is unstable; the obesity rate is rising rapidly, but the exercise intensity is low; and there is a significant gap between policy promotion and behavioral transformation.

**Figure 2:**
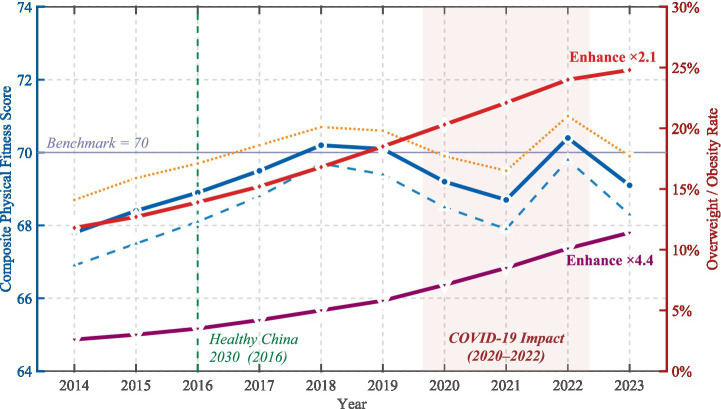
Multi-line trend chart of Chinese college students’ physical fitness indicators over 10 years (2014–2023).

As shown in Figure 2, the comprehensive physical fitness scores of Chinese university students fluctuated around a baseline of 70 points over the past decade, failing to achieve a stable breakthrough. This contrasts sharply with the continued rapid increase in overweight and obesity rates. Notably, the COVID-19 pandemic (2020–2022) did not cause a sharp drop in physical fitness scores, but rather showed some resilience. This may be related to the mandatory implementation of physical education classes and running check-in systems during the lockdown period. However, the obesity rate continued to rise during the same period, indicating that the effectiveness of institutionalized physical activity in maintaining weight is very limited, and the combined effect of individual factors such as high-calorie diets and sedentary lifestyles is more crucial. After the Healthy China 2030 policy milestone (2016), physical fitness scores showed a short-term improvement, but this improvement became unsustainable under the dual pressures of the pandemic and structural factors after 2019, indicating a significant gap between the immediate effects of policy intervention and its long-term sustainability.

### 3.3 Individual-level facilitating and hindering factors

At the individual level, self-efficacy is the most consistent and largest facilitating factor in the current literature. Multiple path analysis studies targeting Chinese university students have reported that the standardized path coefficient of self-efficacy on physical activity participation ranges from 0.35 to 0.46. One study of 2000 Guangzhou university students showed that the direct effect coefficient of self-efficacy on physical activity (*β* = 0.459) was much higher than that of social support (*β* = 0.191) and outcome expectation (*β* = 0.306), and the indirect effect generated through self-regulation further amplified its overall contribution (1). The psychological pathway by which self-efficacy influences physical activity has been confirmed in multiple mechanistic studies: physical exercise can improve emotional regulation ability by enhancing self-efficacy (17), thereby reducing the risk of exercise withdrawal; exercise participation can also positively influence body image satisfaction, self-esteem, and psychological resilience, forming a positive cycle of self-reinforcement (18).

However, academic pressure is currently the most prominent individual-level obstacle faced by Chinese university students. Among the studies included in the analysis, “heavy academic workload” was the most frequently reported obstacle to exercise, especially among college students in their second to fourth years. As academic pressure accumulated, the participation rate in physical activities showed a significant downward trend (19). It is worth noting that academic pressure does not constitute an objective “time constraint,” but rather reflects a subjective sense of resource scarcity. When students regard time and energy as limited resources, physical exercise often ranks after academics in their subjective priorities. This mechanism is particularly prominent among students with low self-efficacy. In terms of gender differences, the proportion of girls participating in organized sports activities is significantly lower than that of boys. This is partly because girls have relatively lower self-efficacy in their athletic abilities and require a higher level of external social support to maintain the same level of participation (20).

### 3.4 Facilitating and hindering factors at the interpersonal level

Interpersonal research evidence suggests that social support networks are an important external catalyst for changes in individual exercise behavior. A path analysis of 604 Chinese university students revealed that physical education teacher support indirectly affects students’ participation in sports through a chain-mediated path of “sports learning motivation → self-efficacy,” with the chain-mediated effect accounting for 47.94% of the total effect (21). Teacher support plays a key “guiding” role in this chain: when teachers provide emotional encouragement and technical feedback, students’ intrinsic motivation for sports learning is enhanced, thereby forming a belief foundation for continuous exercise (22).

A structural equation model study of 1,440 college students found that the effects of school support (*β* = 0.444), peer support (*β* = 0.312), and family support (*β* = 0.145) on physical activity participation show a gradient effect of decreasing order (23), indicating that institutional support (represented by teachers) in the college setting has a stronger behavioral shaping power than informal social support. The peer effect mainly works through two mechanisms: one is the demonstration effect, that is, the imitation motivation generated after observing the exercise behavior of peers; the other is the companionship mechanism, that is, the sense of social belonging brought about by exercising with others. However, this peer effect is bidirectional—when the student group prioritizes academic performance, physical exercise may be collectively labeled as “a waste of time,” creating negative peer pressure.

The role of parents is particularly complex in the Chinese context. On the one hand, parents’ exercise behavior demonstrations have a substantial impact on the formation of students’ early exercise habits (24); on the other hand, the family expectation system centered on academic performance continues to exert a suppressive effect in college, especially in students’ families from rural areas who see higher education as the only path to upward mobility, where this pressure is more pronounced (25). There is a bidirectional inhibitory relationship between mental health problems such as depression and anxiety and physical activity, and the lack of social support networks is an important intermediate variable in this vicious cycle (5).

### 3.5 Organizational facilitators and hinderers

At the organizational level, the design of physical education curriculum has an important structural shaping function on the exercise behavior of college students. Empirical studies based on longitudinal data from the China National Health and Nutrition Survey (CHNS) found that students with physical education classes were 20.2% more likely to exercise independently outside of school than those without physical education classes, and for every 100% increase in physical education class time, the average time for extracurricular physical activity increased by 22.3% (26). This finding reveals the “spillover effect” of physical education courses: formal physical education classes are not only a direct source of physical activity, but can also positively transfer to independent exercise outside of school by strengthening sports skills and establishing exercise habits. However, Chinese universities generally have a system in place where physical education classes for third-year students and above are changed from compulsory to elective, which leads to a significant decline in the participation rate of senior students in physical activities (27), and the cultivation of sports skills and habits is institutionally interrupted in key years.

The impact of the perceived sports environment on students’ exercise benefits is also not negligible. When students have a positive evaluation of the convenience of campus sports facilities, the quality of teacher support, and the sports atmosphere, their enthusiasm for sports participation and their sports self-efficacy are significantly improved (24). However, the construction of campus sports culture is still relatively weak in many colleges and universities: competitive sports culture has high skill requirements and implicitly excludes students with poor sports foundation; the coverage of campus sports clubs and teams is limited and it is difficult to benefit most non-sports major students. The lack of professional sports guidance is another prominent organizational obstacle - the survey shows that only a very small number of colleges and universities are equipped with sports prescription services for all students, and the vast majority of students’ self-training lacks scientific guidance, resulting in low efficiency and high risk of injury (13, 28). Sports policy attitudes also affect students’ physical fitness levels through the mediation of organizations. Related studies have shown that students who have a positive attitude toward school sports policies are more active in their actual exercise behavior and have better physical fitness test scores; this relationship has a clear gender moderating effect, and the behavioral conversion efficiency of policy attitudes is higher among male students (8).

### 3.6 Environmental factors that promote and hinder development

Based on GIS technology, research on campus architectural environment has found that the connectivity of streets within the campus, the degree of land use mixing, and the per capita green space area are all significantly positively correlated with the physical fitness indicators of college students (29). Specifically, in campuses with better walkability, students’ daily physical activity (especially walking and cycling) is significantly higher; green spaces not only provide sports venues but also indirectly reduce the tendency of sedentary behavior by relieving attention fatigue and improving emotional state (26).

**Figure 3:**
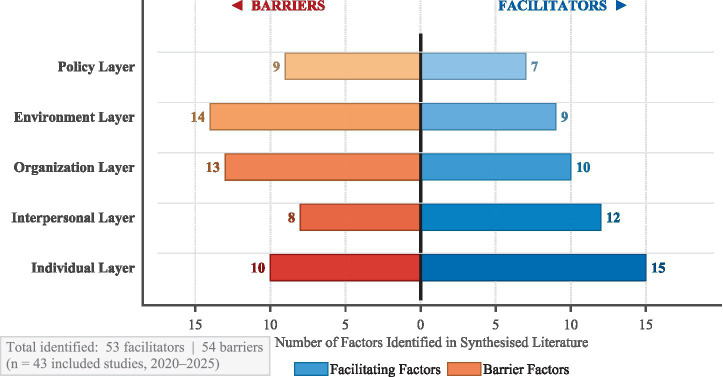
Distribution of facilitating and barrier factors by social-ecological level among Chinese university students’ campus fitness engagement: a synthesis of 43 peer-reviewed studies (2020–2025). Note: Factor counts represent distinct facilitating/barrier items identified across 43 peer-reviewed studies (2020–2025). Data derived from systematic synthesis; color depth reflects level position (darker = proximal). Environment and organization layers show disproportionately higher barriers, warranting priority interventions.

As shown in Figure 3, among the five socio-ecological levels, the number of hindering factors identified at the environmental level (14 items) is significantly higher than the number of promoting factors (9 items), showing the largest “promotion-hindering asymmetry” among all levels. This pattern reveals the urgency of improving the physical environment of the campus. Data from the Sixth National Sports Venue Census shows that at the end of 2013, the per capita sports venue area in China was only 1.46 square meters, less than one-tenth of that in Japan (19.2 square meters); among the completed venues, only 51.5% are fully open to the public, and about 34.2% are membership-based fee venues (30). According to the 2024 National Sports Facilities Statistical Survey, by the end of 2024, the total number of sports facilities in China had increased to 4.8417 million, with the per capita area increasing to 3.0 square meters (31). However, the gap in the allocation of facilities resources between the eastern and western regions remains very prominent. Figure 3 further reveals that there is a clear advantage of obstacles at the organizational level (13 obstacles vs. 10 promotions), while at the individual and interpersonal levels, promotion factors dominate (15: 10 and 12:8, respectively). This means that intrinsic psychological motivation and social networks are relatively favorable intervention leverage points, while facilities and institutional arrangements are currently the weakest supportive environment. In the context of universities, problems such as crowded facilities, limited opening hours (especially at night or on weekends), and high fees for some facilities have appeared in many qualitative studies, constituting the main external obstacles to students’ independent exercise after class (12, 13, 32). Exploring the effective use of facility resources through the university-community joint use agreement (JUA) is a realistic and feasible improvement path (19). Based on the analysis of the large-scale longitudinal data of the China Education Panel Survey (CEPS), the hierarchical linear model shows that the three environmental microsystems of family, community and school have independent positive predictive effects on adolescent physical activity behavior, and the quality of family-school relationship has a moderating effect on the impact of school environment (24, 28, 33). This means that the effectiveness of the campus physical environment depends on the cooperation between family and school.

### 3.7 Policy-related factors promoting and hindering progress

The evidence from the policy level presents a picture full of tension. On the one hand, China has formed a relatively complete fitness promotion policy system. The “Healthy China 2030” plan (2016) lists national fitness as a core national strategy, and the National Fitness Program (2021–2025) clearly proposes to build a multi-level fitness public service system. The “National Student Physical Fitness and Health Standard” provides an operable student physical fitness assessment framework for colleges and universities (5, 15). On the other hand, the empirical evaluation of policy effects reveals systemic gaps. International comparative studies show that the physical activity level of Chinese children and adolescents aged 9–17 ranks 53rd among 57 surveyed countries and regions in the latest round of the 2016–2022 series of global matrix assessments, with an overall average grade of D (34). Although the data study focused on school-aged children and adolescents rather than university students, this macro-policy performance evaluation reveals a systemic gap in China’s physical activity promotion policies across the entire life cycle. This echoes and corroborates the insufficient participation observed during the university stage in this study, indicating that the root cause of the problem has institutional characteristics that span across age groups.

The structural reasons for this policy implementation gap are mainly reflected in three dimensions: First, the policy objectives are directed at the macro total (such as the total number of sports facilities and the number of social sports instructors) rather than the precise promotion of behavioral levels; Second, there is a huge gap in the allocation of sports resources between universities in the east and west, and the facilities and teachers of some western universities are far from reaching the baseline set by the policy; Third, the institutional inertia of “separation of sports and education” still exists, and the sports authorities and education authorities lack effective coordination in the formulation and implementation of university sports policies, resulting in sports policies remaining at the document level and being difficult to effectively embed into daily teaching management (2, 16).

### 3.8 Comprehensive comparison of the relative importance of factors at each level

The comprehensive effects of the five social ecological levels are summarized in Table 5. In terms of effect size, self-efficacy at the individual level consistently exhibits a medium to large effect (*β*: 0.35–0.46), with the strongest evidence. Teacher support at the interpersonal level has a moderate effect size (*β*: 0.12–0.22), but its indirect influence through mediation mechanisms should not be underestimated. The direct effect size at the organizational and environmental levels is relatively small, but these two levels are the “underlying structures” that constrain the effectiveness of other levels, and their improvement has an amplifying effect. The quality of evidence at the policy level is limited by the heterogeneity of measurement tools, and the estimation of direct effects is still unstable.

**Table 5:** Summary of comprehensive effects of facilitating and barrier factors across social-ecological levels.

Level	Key facilitating factors (representative effect sizes)	Key barrier factors	Evidence strength	Intervention priority
Individual	Self-efficacy (*β* = 0.35–0.46); exercise motivation; positive outcome expectations	Academic pressure; low self-efficacy; exercise anxiety	Strong	High
Interpersonal	Teacher support (*β* = 0.12–0.22; chain mediation 47.94%); peer companionship	Academic-oriented parental expectations; lack of exercise partners	Moderate–strong	High
Organizational	Mandatory PE curriculum (+20.2% extracurricular exercise); sports clubs	Elective PE for upper-year students; lack of professional guidance	Moderate	Moderate–high
Environment	Facility accessibility; green space area; pedestrian connectivity	Overcrowded/fee-charging facilities; limited opening hours; urban–rural gap	Moderate	Moderate
Policy	Healthy China 2030; National Fitness Plan; fitness standard incentives	Insufficient implementation monitoring; resource inequality between eastern and western regions	Weak–moderate	Moderate–low

## 4 Discussion

### 4.1 Self-efficacy and exercise motivation: core intrinsic drivers at the individual level

Based on the effect size estimates of multiple studies, self-efficacy is the most robust and consistent predictor of physical activity reported in the literature to date. This conclusion spans Chinese university students from different regions and with different professional backgrounds, and remains significant even after controlling for objective environmental factors (23). From a mechanistic perspective, students with high self-efficacy are not only more inclined to initiate exercise behavior, but also more able to maintain exercise persistence when faced with obstacles such as academic pressure and insufficient facilities, demonstrating stronger behavioral resilience (35). Interventions targeting self-efficacy are most defensibly grounded in Bandura’s Social Cognitive Theory, which specifies four primary sources of efficacy belief that should be simultaneously addressed in program design, namely mastery experiences through graduated sports skills training, vicarious learning through peer demonstration, verbal persuasion through teacher and peer feedback, and physiological state management through the regulation of anxiety and fatigue associated with exercise. Theoretically anchored interventions of this kind possess a leverage value of using a focal intervention point to drive whole-system change and can offset, to a measurable extent, the negative influence of external environmental obstacles.

However, a reverse causal relationship that is easily overlooked deserves attention: the weakening effect of academic pressure is not simply achieved through “time occupation,” but is more reflected in the cognitive compression of the subjective importance of physical activity. When students allocate time as a scarce resource, physical exercise is systematically disadvantaged in the value ranking. This “cognitive time competition” exists even when actual available time is not scarce. This mechanism suggests that simply increasing the supply of time is not enough to effectively improve participation in physical activities. Changing students’ subjective cognitive framework of the value of exercise is the core entry point for intervention (17, 18). It is worth noting that gender differences are particularly prominent at the individual level: female students not only have generally lower baseline self-efficacy, but also need to obtain a higher level of external social support than male students to maintain the same level of participation (20). This suggests that intervention programs for female college students need to work on both individual beliefs and social support at the same level, and should not mechanically apply general intervention strategies to this subgroup (36).

### 4.2 Teacher-student and peer relationship network: interpersonal social incentive mechanism

Evidence at the interpersonal level shows that the mechanism of physical education teacher support is far more complex than a simple “incentive effect.” In the chain mediation framework, teacher support first activates students’ intrinsic motivation for physical education, then strengthens their self-efficacy in sports, and finally transforms into sustained participation behavior (21). This pathway carries an important policy implication, namely that the core function of physical education teachers should not be limited to skill instruction but should extend to the stimulation of sports motivation and the cultivation of efficacy belief. Traditional skill-oriented physical education classes are insufficient for this broader mandate and require transformation into an autonomy-supportive teaching model (37). Teacher professional development should accordingly incorporate training in emotional intelligence and emotional regulation competencies, given the documented association between emotional regulation capacity and sustained exercise participation operating through the self-efficacy pathway (17), and the embedding of emotional regulation content within the physical education curriculum is recommended as a structural vehicle for transmitting these competencies to students.

The two-way nature of the peer effect also needs to be understood dialectically in the context of Chinese culture. On the one hand, peer demonstrations and companionship can effectively reduce the psychological cost of “exercising alone” and enhance the sense of belonging to sports; on the other hand, when dormitory culture or class norms dominated by academic competition are prevalent, students often regard the “exposure” of exercise time as a signal of non-sociality, thus resulting in social withdrawal (13, 38). This “double-edged sword” effect suggests that university intervention strategies should not target individual behavior in isolation, but should take the dormitory or class as a whole intervention unit and activate the positive peer effect by changing micro-social norms.

The influence of parents’ sports behavior demonstrations on children’s habit formation has not disappeared after entering university, but its form of action has changed from direct supervision to behavioral demonstration and transmission of sports values (24). In most Chinese families where academic performance defines success, the “instrumental value” of exercise—such as improving learning efficiency and mental state—is more readily accepted by parents than its “intrinsic value,” thus translating into substantial support for their children’s exercise behavior.

### 4.3 Physical education curriculum and campus sports culture: institutional factors at the organizational level

The core finding at the organizational level is that the power of institutional design far outweighs the power of simple resource input. The “spillover effect” of physical education classes—that is, students who take physical education classes are also more active outside of class—is not a coincidence, but a byproduct of the curriculum establishing exercise skills and habits (16). This mechanism has direct evidence-based implications for university physical education curriculum policies: extending physical education coverage to the third and fourth years, or replacing mandatory compulsory arrangements with elective course credit incentives, may be a low-cost and high-efficiency path to increase the participation rate of senior students in physical activities.

However, the number of obstacles at the organizational level (13 items) exceeded the number of facilitators (10 items) in this comprehensive analysis, indicating that the current organizational system of Chinese universities is still biased toward consuming exercise habits rather than cultivating them. This judgment is highly consistent with the conclusions of existing systematic reviews—the lack of professional sports guidance, the narrow coverage of sports clubs, and the exclusionary effect of competitive culture on ordinary students constitute structural obstacles at the organizational level (28, 39). In addition, meta-analysis studies of sports interventions have shown that campus intervention programs that include multiple forms of sports and last for more than 12 weeks have significant effects on improving various indicators of physical fitness standards (40). However, the implementation and promotion of such programs in Chinese universities still face practical constraints such as insufficient teachers and limited class hours. The perceived quality of the sports environment is also worth noting. When students’ overall perception of campus sports facilities, curriculum design, and sports atmosphere tends to be positive, their sports self-efficacy and sense of gain from sports are significantly improved (24). This means that improvements at the software level (atmosphere creation, cultural construction) and at the hardware level (facility construction) have equally important intervention value, while the former is often overlooked in policy discussions.

### 4.4 Venue facilities and architectural environment: the hardware foundation at the environmental level

The obstacles at the environmental level are the most prominent in this comprehensive analysis, which is consistent with the conclusions of existing reviews targeting university students (12, 20). The per capita sports venue area increased from 1.46 square meters in 2013 to 3.0 square meters in 2024 (26, 31), an increase of approximately 105%, but still significantly lower than that of high-income countries. While this increase is considerable, it still lags behind the levels of high-income countries, and the differences in the quality and accessibility of existing facilities also constrain actual utilization efficiency (31).

Objective measurement data of the campus architectural environment reveals a more refined influence mechanism: different types of architectural environment characteristics have differentiated effects on different forms of physical activity (5). Pedestrian connectivity mainly affects daily physical activity, green space area has a more significant impact on independent leisure exercise, while the density and quality of specialized sports facilities mainly affect purposeful exercise behavior. This means that “increasing facilities” is not a single, homogeneous intervention logic, but requires differentiated environmental design based on the target behavior type (32).

The venue sharing mechanism provides a feasible path to overcome the constraints of campus resources. The pilot practice of the Joint Use Agreement (JUA) model between universities and communities in cities such as Nanjing shows that, under the premise of ensuring campus management safety, the appropriate opening of university venues to the public can not only improve the efficiency of resource utilization, but also enhance the synergistic effect of sports atmosphere between universities and surrounding communities (19), indirectly creating a richer selection of sports venues for university students.

### 4.5 Policy supply and implementation gap: institutional barriers at the macro level

From the perspective of policy development history, China’s sports and health policies have undergone three stages of evolution from ideological mobilization, legal norms to systemic health governance (34). “Healthy China 2030” marks an important turning point in this process—repositioning sports from the traditional “educational function” to a “health strategy tool,” establishing the public health attributes of university sports at the top-level design level (41). However, the gap between policy intent and implementation results is clearly visible in the macro-assessment data: the physical activity level of Chinese school-age children and adolescents (9–17 years old) has long been at the bottom of the global matrix rating (31) - it should be noted that this data comes from the school-age sample, which is different in age from the college student group in this study; however, considering the continuity of physical activity behavior trajectory, the low participation pattern in the school-age stage often continues into the university stage (3), and the two share the same policy and institutional background. Therefore, this data still has reference value at the policy assessment level, which shows that the perfection of the policy text is not equivalent to the policy implementation effect, and the institutional transformation capacity is the key variable that determines the policy effectiveness.

The deep-seated cause of policy-level obstacles lies in the problem of “incentive compatibility” - the current college assessment system systematically attaches less importance to the quality of physical education teaching than to academic indicators, and the career development path of physical education teachers is relatively limited, which makes the motivation for improving the sports system at the organizational level insufficient. The persistent gap in sports resource allocation between universities in the east and west further exacerbates the failure of market-driven or competitive policies in underdeveloped regions, necessitating the precise integration of special financial support and regional balance policies (42).

### 4.6 Cross-level interaction mechanisms and intervention priorities

The cross-level analysis in this synthesis suggests that the relationship between factors at each level is not a simple linear superposition but rather reflects a hierarchical associational structure. Evidence from the multi-level structural equation analysis reported by Ge et al. (10) indicates a directional pattern consistent with an environment–organisation–interpersonal–individual configuration, although the predominantly cross-sectional nature of the included studies and the reliance on subject variables rather than experimentally manipulated exposures preclude definitive causal attribution. The policy implication of this pattern is that, in addition to psychological intervention at the individual level, the improvement of remote environmental and institutional factors has an undeniable structural value: even if self-efficacy is improved in the short term through psychological intervention, if supporting facilities, institutions, and social norms are continuously lacking, this improvement in efficiency is difficult to translate into sustainable behavioral change. Meanwhile, the interpersonal level exhibits a relatively symmetrical two-way interactive relationship with the individual level—the improvement of individual self-efficacy can in turn promote the construction of positive social relationships (such as actively seeking sports partners and participating in sports communities), forming a positive reinforcement cycle. This mechanism suggests that the integrated implementation of individual psychological intervention and peer social intervention may have a synergistic gain effect that surpasses single-level intervention (43).

Drawing together the level-specific evidence summarized in Table 5, the prioritization of self-efficacy cultivation and teacher support as immediate intervention entry points is justified by the convergence of three considerations, namely the magnitude and consistency of the reported effect sizes, the methodological strength of the underlying evidence, and the feasibility of short-horizon implementation within existing university governance structures. Self-efficacy cultivation is supported by the strongest evidentiary base, with standardized path coefficients ranging from 0.35 to 0.46 across multiple independent samples, and can be operationalized through existing curricular and extracurricular channels at low marginal cost. Teacher support interventions rest on established chain-mediated pathways in which exercise motivation and self-efficacy jointly transmit the effect of teacher behavior on sustained participation, and can be delivered through moderate professional-development investment with a favorable effect-to-cost profile. These immediate priorities should not be mistaken for substitutes for structural reform, because in the absence of parallel improvement of organizational and environmental conditions single-level interventions are expected to encounter ceiling effects as documented in the broader implementation literature. Accordingly, the priority of intervention at each ecological level is articulated in Table 6, which specifies the core facilitators, barriers, short-term and long-term recommendations, responsible parties, and intervention priority for each level.

**Table 6:** Intervention recommendations and priority matrix across social-ecological levels.

Level	Core facilitating factors (to strengthen)	Core barrier factors (to eliminate)	Short-term intervention recommendations	Long-term intervention recommendations	Responsible party	Priority
Individual	Self-efficacy; intrinsic exercise motivation	Academic pressure; exercise anxiety	Self-efficacy cultivation workshops grounded in Social Cognitive Theory; integrated mental health and exercise curriculum	Embed physical health literacy into curricula	Universities (psychology / PE departments)	High
Interpersonal	Teacher support; peer companionship	Academic-first family norms	Autonomy-supportive PE teaching reform; teacher training in emotional intelligence; peer exercise groups	Family health education linkage mechanisms	University teachers; student advisors	High
Organizational	Full-year PE courses; sports clubs	Upper-year elective PE policy; lack of professional guidance	Extend mandatory PE coverage; introduce exercise guidance specialists	Diversify competitive sports culture; establish campus sports credit system	University academic affairs/PE departments	Moderate–High
Environment	Facility accessibility; green spaces	Overcrowding; fees; limited opening hours	Extend facility opening hours; introduce reservation and flow management systems	Standardize campus built environment; promote JUA facility-sharing mechanisms	University facilities management; local government	Moderate
Policy	Healthy China 2030; National Fitness Plan	Sport–education separation; resource inequality	Establish precise performance evaluation indicators for university PE quality	Institutionalize sport–education coordination; earmark funds for east–west regional equity	Ministry of Education; General Administration of Sport	Moderate

### 4.7 Research limitations and future directions

This study has several methodological limitations that need to be acknowledged. The provincial representativeness of the publicly available statistical data varies considerably, and this constrains the external validity of the quantitative trend estimates. The two large longitudinal datasets utilized in the present synthesis originate from Anhui Province (*n* = 58,472) and Shandong Province (*n* = 176,373), both located in the eastern and central regions, and therefore cannot fully capture the heterogeneity of universities across the eastern, central, and western regions of China, particularly the resource-constrained conditions documented in western higher-education institutions. Some data releases are subject to time lags, which may introduce discrepancies with current realities. Additional interpretive caution is warranted when drawing on policy-level comparative indicators such as the global matrix assessment results, as these benchmarks are derived from samples of school-aged children and adolescents (9–17 years) rather than university students, and are employed here as macro-contextual references rather than as direct measurements of university-student behavior. While narrative synthesis methods offer flexibility, they cannot rigorously merge effect sizes across studies, and the heterogeneity of measurement tools among studies also limits the accuracy of quantitative comparisons. Furthermore, the measurement of policy-level impacts still lacks universally accepted standardized indicators, resulting in the weakest evidence strength at this level.

Future research should advance in the following directions: conducting longitudinal tracking studies covering five levels of factors in the university setting to establish a stronger foundation for causal inference; conducting multi-level intervention experiments with randomized or quasi-randomized designs to examine the decomposable effects of intervention components; strengthening comparative analyses based on urban–rural stratification and university type stratification (key universities versus vocational colleges); focusing on the embedded effects of digital health technologies (sports apps, wearable devices, campus fitness platforms) at the organizational and individual levels, and the reshaping effect of post-pandemic lifestyles on the impact patterns at various levels.

The findings of the present synthesis are situated within a distinct sociocultural and institutional context that bounds their external generalisability. Chinese university settings are shaped by Confucian-influenced educational values that prioritize academic achievement, hierarchical teacher–student relationships that amplify the behavioral influence of teacher support, and collectivist peer dynamics under which dormitory-based and class-based norms exert strong shaping effects on individual behavior. The centralized national policy architecture, the mandatory physical education curriculum structure, and the residential campus configuration further distinguish Chinese universities from most higher-education systems in Western or mixed-model contexts. Consequently, the relative magnitudes of factors identified at the interpersonal and organizational levels, as well as the salience of the top-down associational pattern observed across levels, should be interpreted as culturally contingent rather than universally applicable. Comparative cross-cultural research is warranted to delineate which components of the multilevel evidence base transfer across educational systems and which remain specific to the Chinese institutional environment.

In addition, some macro-comparative data at the policy level in this study (such as the results of the global matrix assessment) are derived from a sample of school-aged children and adolescents (9–17 years old), which differs from the age group of college students (18 years and older) who are the core focus of this study. This study uses them as macro-background indicators of policy performance rather than as a direct measurement of the physical activity level of college students. Readers should pay attention to this extrapolation boundary when interpreting the relevant conclusions.

## 5 Conclusion

This study uses the social ecology model as a theoretical framework, integrates 43 peer-reviewed articles and multiple national open databases, and systematically sorts out the promoting and hindering factors of college students’ participation in fitness at five levels: individual, interpersonal, organizational, environmental and policy. It also constructs a comprehensive evidence system that reflects the relative importance and interactive transmission mechanism of each level. The study shows that self-efficacy is the most robust individual-level promoting factor, teacher support is the core lever at the interpersonal level, the physical education curriculum system is the key variable at the organizational level, the accessibility of venues is the main obstacle at the environmental level, and the policy implementation gap is a structural problem that urgently needs to be addressed at the macro level. Facilitating and hindering factors do not operate in isolation at each level but rather appear to reinforce or attenuate one another through a multi-level associational pattern running from environment through organisation and interpersonal relations to individual psychology, an interpretive pattern that underscores the limitations of single-level intervention while remaining conditional upon future longitudinal and experimental confirmation. Improving the current state of fitness participation among Chinese university students relies on a systematic approach that coordinates five levels, rather than any single-dimensional policy enhancement. Among these, cultivating self-efficacy (individual level) and strengthening teacher support (interpersonal level) should be prioritized as immediate intervention points, while institutional reform (organizational level) and improvement of the built environment (environmental level) should be considered as structural breakthroughs for systemic improvement. The former is quick to take effect but depends on external conditions, while the latter has a far-reaching impact but a longer cycle. The synergistic advancement and mutual support of these two approaches represent the most cost-effective multi-level intervention configuration logic at present.

## Data Availability

The original contributions presented in the study are included in the article/Supplementary material, further inquiries can be directed to the corresponding author/s.
