# Supplementary material for: A social-ecological analysis of facilitators and barriers to campus fitness engagement: multilevel evidence from Chinese universities
Source: Front Public Health. 2026 Jun 4;14:1856173. doi: 10.3389/fpubh.2026.1856173 (PMC13275693; doi:10.3389/fpubh.2026.1856173)
Supplement: Supplementary file 1 [file Table_1.DOCX]

# **Appendix A List of 43 Included Studies**

| **No.** | **First Author (Year)** | **Journal / Source** | **Study Type** | **SEM Layer** |
| --- | --- | --- | --- | --- |
| **Category A: Individual Layer（n=8）** | | | | |
| A1 | Zhang Y et al. (2022) | Front Psychol | Cross-sectional | Individual |
| A2 | Niu X et al. (2025) | Front Psychol | Cross-sectional | Individual |
| A3 | Zhang S (2025) | Front Psychol | Cross-sectional | Individual |
| A4 | Ouyang Y et al. (2020) | Front Psychol | Cross-sectional | Individual |
| A5 | Mu FZ et al. (2024) | PLoS One | Cross-sectional | Individual |
| A6 | Li L et al. (2023) | Sci Rep | Cross-sectional | Individual |
| A7 | Sun J et al. (2024) | PLoS One | Cross-sectional | Individual |
| A8 | Luo L et al. (2023) | J Clin Med | Cross-sectional | Individual |
| **Category B: Interpersonal Layer（n=7）** | | | | |
| B1 | Su W & Liu Q (2025) | Front Psychol | Cross-sectional | Interpersonal |
| B2 | Liu X et al. (2021) | Think Skills Creat | Cross-sectional | Interpersonal |
| B3 | Zhao X et al. (2025) | Sci Rep | Longitudinal | Interpersonal/Environment |
| B4 | Peng L et al. (2022) | Sustainability | Cross-sectional | Interpersonal |
| B5 | Hu D et al. (2025) | Front Psychol | Systematic Review | Interpersonal |
| B6 | Zhou J & Liu C (2025) | Front Public Health | Narrative Review | Interpersonal |
| B7 | Ge S et al. (2025) | Front Psychol | SEM Cross-sectional/SEM | Interpersonal |
| **Category C: Organization Layer（n=9）** | | | | |
| C1 | Pan M et al. (2022) | Int J Environ Res Public Health | Systematic Review | Organization |
| C2 | Wang D et al. (2024) | J Sch Health | Longitudinal (CHNS) | Organization |
| C3 | Zhao H et al. (2024) | Front Psychol | Cross-sectional | Organization |
| C4 | Song D et al. (2024) | Curr Psychol | Cross-sectional | Organization |
| C5 | Dai S et al. (2025) | Front Educ | Scale Development | Organization |
| C6 | Sun J et al. (2024) | Front Physiol | Meta-analysis | Organization |
| C7 | Hu D et al. (2021) | Int J Environ Res Public Health | Systematic Review | Organization |
| C8 | Ferreira Silva RM et al. (2022) | PLoS One | Systematic Review | Organization |
| C9 | Ames KN et al. (2024) | BMC Public Health | Systematic Review | Organization |
| **Category D: Environment Layer（n=9）** | | | | |
| D1 | Li X et al. (2022) | Int J Environ Res Public Health | Cross-sectional (GIS) | Environment |
| D2 | Wang K et al. (2024) | Front Public Health | Narrative Review | Environment |
| D3 | Wang K & Wang X (2020) | Int J Environ Res Public Health | Policy Analysis | Environment |
| D4 | Sun Y et al. (2024) | J Clean Prod | Scenario Analysis | Environment |
| D5 | Dong D et al. (2025) | Front Public Health | Policy Analysis | Environment |
| D6 | Zhao X et al. (2025) | Sci Rep | Longitudinal | Environment/Interpersonal |
| D7 | Liang F et al. (2025) | BMC Public Health | Longitudinal | Environment |
| D8 | Zhang M et al. (2025) | BMC Public Health | Cross-sectional | Environment |
| D9 | Cai S et al. (2025) | Lancet Reg Health West Pac | Longitudinal (5 national surveys) | Environment |
| **Category E: Policy Layer（n=10）** | | | | |
| E1 | Gao G et al. (2024) | Front Public Health | Historical Review | Policy |
| E2 | Chen B et al. (2025) | Front Public Health | Policy Analysis | Policy |
| E3 | Cao ZB et al. (2023) | Humanit Soc Sci Commun | Policy Analysis | Policy |
| E4 | Cao ZB et al. (2022) | J Exerc Sci Fitness | Report Card Analysis | Policy |
| E5 | Wang S & Brownson RC (2020) | J Sport Health Sci | Policy Analysis | Policy |
| E6 | WHO (2022) | Global Status Report | Official Report | Policy |
| E7 | Bull FC et al. (2020) | Br J Sports Med | Guideline | Policy |
| E8 | van Sluijs EMF et al. (2021) | Lancet | Review | Policy |
| E9 | Recchia F et al. (2023) | JAMA Pediatr | Systematic Review & Meta-analysis | Policy |
| E10 | Sallis JF et al. (2015) | Health Behavior (Book Chapter) | Theoretical Framework | Policy |

***Notes：***

*1. 47 included studies are grouped by SEM level: Individual (n=10), Interpersonal (n=9), Organization (n=9), Environment (n=9), Policy (n=10).*

*2. Studies marked with dual layers span two SEM levels; classification reflects the primary theoretical contribution.*

*3. Study codes correspond to in-text citation numbers in square brackets.*

*4. Studies marked 'Unpublished' refer to conference reports or internal datasets; source details are provided in the main text.*

**Appendix Figure S1.** PRISMA 2020 Flow Diagram for Literature Search and Screening.

*Note: Note Longitudinal/Quasi-experimental (n=9): longitudinal n=6, quasi-experimental n=3. Systematic reviews/Policy analysis (n=7): systematic reviews n=5, policy analyses n=2. The search period spans from January 2020 to June 2025, covering four databases: PubMed, Web of Science, Scopus, and CNKI. A total of 47 peer-reviewed articles were ultimately included for systematic synthesis.*
